# Giant Parathyroid Tumor: Parathyroid Adenoma versus Parathyroid Carcinoma

**DOI:** 10.1155/2022/7712097

**Published:** 2022-10-26

**Authors:** Farid Gossili, Allan Carlé, Trine B. Andersen, Helle D Zacho

**Affiliations:** ^1^Department of Nuclear Medicine, Aalborg University Hospital, Aalborg, Denmark; ^2^Department of Endocrinology, Aalborg University Hospital, Aalborg, Denmark; ^3^Department of Clinical Medicine, Aalborg University, Aalborg, Denmark

## Abstract

Parathyroid adenoma is the most common cause of primary hyperparathyroidism (PHPT). We present the preoperative detection of a giant parathyroid adenoma (GPA) using (^99m^Tc)-sestamibi parathyroid scintigraphy in a patient presenting with severely elevated parathyroid hormone, hypercalcemia, hypophosphatemia, and vitamin D insufficiency. The patient complained of cerebral symptoms and intermittent abdominal discomfort without constipation. After surgical removal of the hyperactive parathyroid gland and D vitamin supplementation, all blood tests were normalized. The clinical and paraclinical characteristics of GPA may raise the suspicion of parathyroid carcinoma, but not absolutely in this case.

## 1. Introduction

Primary hyperparathyroidism (PHPT) has an incidence of 0.4 to 82 cases per 100,000 person/year and is characterized mainly by elevated parathyroid hormone (PTH) levels and, consequently, hypercalcemia and hypophosphatemia [1]. The most common cause of PHPT is a solitary parathyroid adenoma (80–85%) [1, 2]. A very enlarged parathyroid adenoma is a rare variation in which the adenoma weighs more than 3500 mg and is known as a giant parathyroid adenoma (GPA) [3, 4]. In addition to clinical complications such as very high levels of calcium and PTH and possible mass effects, GPA can also cause operative challenges during surgical resection [1]. On the other hand, parathyroid carcinoma accounts for approximately 1% of PHPT [1, 2], and suspicion of parathyroid carcinoma increases with increasing adenoma size [5]. Therefore, differentiation with parathyroid carcinoma is very important in cases of giant parathyroid adenoma.

Parathyroidectomy is recommended as the definitive treatment of parathyroid adenoma in cases of young age, high total serum calcium, osteoporosis, renal calcifications, or the presence of symptoms [6]. Minimally invasive parathyroidectomy is considered the best method in terms of reducing surgical complications as well as the cost and length of hospitalization. Consequently, preoperative localization of parathyroid adenoma is very important to allow for minimally invasive surgery [1]. Preoperative diagnosis of parathyroid carcinoma is impossible in the vast majority of patients [7], although very high PTH and calcium levels, as well as palpable tumors and a large amount of tissue, may suggest parathyroid carcinoma. However, due to a lack of preoperative diagnostic modalities, the majority of parathyroid carcinomas are diagnosed postoperatively using pathologic examinations [8].

Parathyroid scintigraphy with (^99m^Tc)-sestamibi is the gold standard for preoperative localization of the parathyroid tissue, according to some studies [9]. However, several studies have shown that imaging accuracy increases when scintigraphy is combined with ultrasound [1, 10].

The combination of a giant parathyroid mass seen on (^99m^Tc)-sestamibi scintigraphy and ultrasound, along with severely elevated PTH levels, may raise suspicion for parathyroid carcinoma. This case report is based on a patient presenting all those features.

## 2. Case Report

A 38-year-old Caucasian woman, previously healthy, was referred to an endocrinology outpatient clinic due to persistent hypercalcemia for one year as well as elevated PTH levels, mild hypophosphatemia, and vitamin D insufficiency. She had psychiatric moans and abdominal discomfort without constipation. She had no familial predisposition to multiple endocrine neoplasias 1 (MEN1). Laboratory tests showed a serum calcium level of 2.93 mmol/l (reference range 2.20–2.55 mmol/l), serum albumin-corrected calcium level of 2.88 mmol/l (reference range 2.20–2.55 mmol/l), serum phosphorus of 0.66 mmol/l (reference range 0.76–1.41 mmol/l), serum 25-hydroxyvitamin D of 17 (reference range 50–160 nmol/l), and a PTH level of 52 pmol/l (reference range 1.3–7.6 pmol/l). The calcium/creatinine clearance ratio was 0.013, which may indicate both familial hypercalcemia hypocalciuric (FHH) and primary hyperparathyroidism [11]. However, gene analysis (CASR, calcium-sensing receptor) was negative for FHH, and primary hyperparathyroidism due to parathyroid adenoma was suspected. CT urography revealed no nephrocalcinosis. Dual-energy X-ray absorptiometry detected osteopenia (T-scores of −2.4/−1.8 in the lower back/femoral neck, respectively).

**Figure 1:**
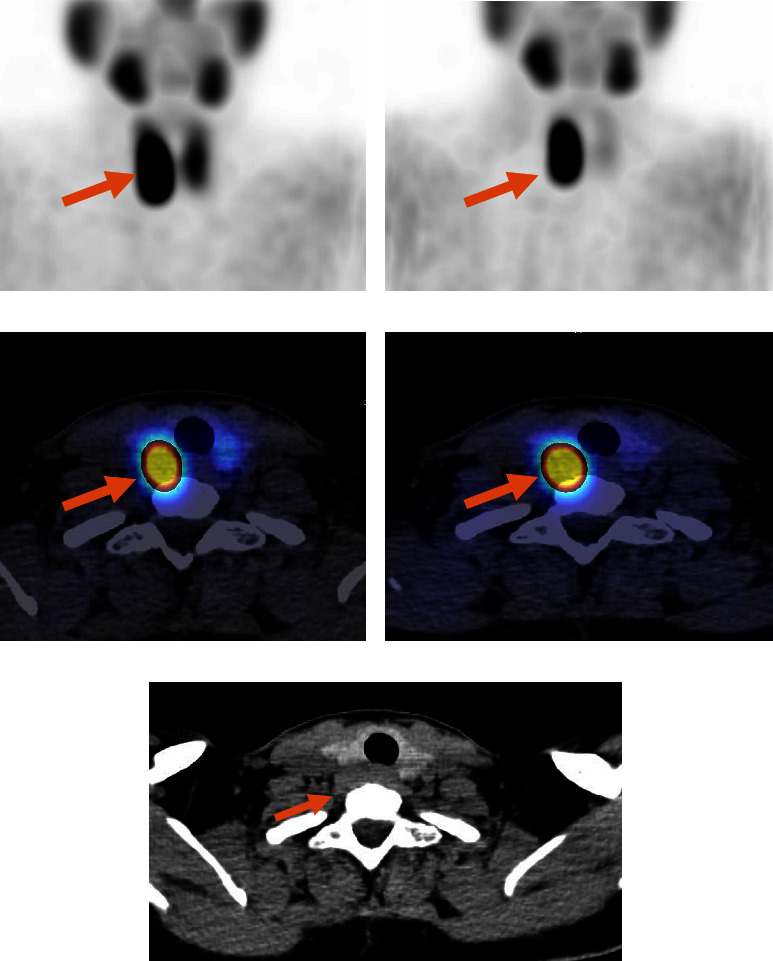
A 38-year-old woman with a giant parathyroid adenoma, localized by a sestamibi scan. Immediate (a) and delayed (2-hour) (b) anterior images of (^99m^Tc)-sestamibi demonstrate a focus of intense sestamibi uptake (arrows). Immediate (c), delayed (2-hour) (d) axial fused SPECT/CT images, and transverse noncontrast CT image (e) localized a circular area with focal uptake posterior to the right thyroid lobe (arrows), which was confirmed histologically to be a giant parathyroid adenoma.

**Figure 2:**
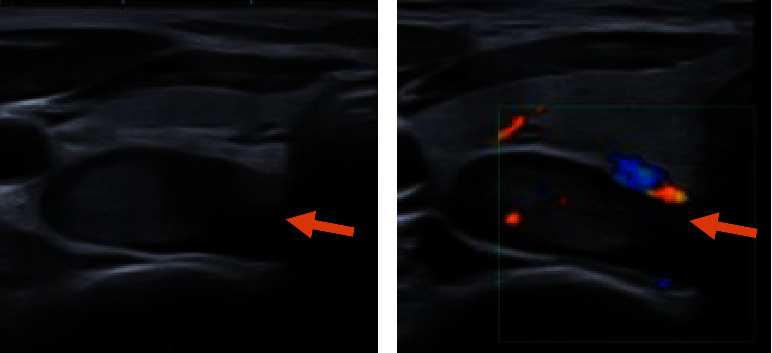
The ultrasound from the patient with a giant parathyroid adenoma. Transverse grayscale images (a) show a giant parathyroid adenoma with a thick capsule and internal echogenic components (arrow). Transverse color doppler images (b) show minimal detectable internal flow (arrow).

The patient underwent parathyroid scintigraphy aiming for preoperative localization of the possible parathyroid tumor. Dual-phase (^99m^Tc)-sestamibi scintigraphy with single-photon emission computed tomography (SPECT) detected a large parathyroid tumor measuring 38 × 18 × 14 mm (craniocaudal x transverse x anteroposterior) located behind the right thyroid lobe compatible with a giant parathyroid adenoma (Figure 1). An ultrasound scan confirmed the scintigraphically detected tissue (Figure 2). The severely elevated PTH levels in this young patient raised the suspicion of a possible parathyroid carcinoma. However, the only slightly increased calcium level indicated otherwise.

The patient underwent minimally invasive parathyroidectomy, and PTH levels (from 34.4 to 3.6 pmol/l), and calcium levels normalized both preoperatively and during follow-up. Vitamin D insufficiency was treated with vitamin D supplementation and serum vitamin D near-normalized during follow-up (from 17 to 47 nmol/l). The postoperative parathyroid adenoma weighed 4 grams. Histopathology revealed a rounded structure with a 1 mm thick connective tissue capsule. The structure consisted of confluent trabecular formations of chief cells with eosinophilic vesicular cytoplasm and relatively small, hyperchromatic nuclei. These changes are representative of a sporadic parathyroid adenoma.

## 3. Discussion

Giant parathyroid adenoma is a rare cause of PHPT, and consequently, only a few cases of GPA have been reported. A systematic review by Wong et al. identified 62 GPAs from 1952 to 2021 [3]. Reporting a very rare case, namely this case, is important both paraclinically and clinically, as it can contribute to the assessment of this type of patient in possibly challenging situations.

Preoperative differentiation between giant parathyroid adenoma and parathyroid carcinoma is very challenging. The preoperative clinical and paraclinical presentation of giant parathyroid adenoma, especially with severely elevated PTH and calcium levels, may be very similar to that of parathyroid carcinoma [3]. A familial predisposition to MEN1, severely elevated serum corrected calcium level (>3 mmol/l) with several clinical symptoms of hypercalcemia, including nausea, vomiting, abdominal pain, constipation, myopathy and neurocognitive deficit, very high PTH levels (5–10 times more than the upper limit of the reference range), renal and bone involvement, very large tissue and younger age at presentation, can raise the suspicion of parathyroid carcinoma [12–17]. In this case, there was a giant tumor with severely elevated PTH levels in a young woman; however, negative familial disposition to MEN1 only slightly increased corrected calcium levels and no signs of nephrolithiasis or osteoporosis. These characteristics indicate parathyroid adenoma rather than carcinoma. This case demonstrates the importance of a high calcium level with concomitant increasing PTH levels and an enlarged tumor in the preoperative suspicion of parathyroid carcinoma.

According to the international consensus on the management of PHP [18], the preoperative localization of tumor in the parathyroid glands is very important in planning the operation and determining the proper method, especially in minimally invasive surgery. However, the standard of care in the treatment of parathyroid carcinoma is ‘‘en bloc” resection of the carcinoma with ipsilateral hemithyroidectomy and reoperation in some patients [8]. Therefore, the differentiation between PTA and parathyroid carcinoma has clinical importance for the choice of the correct surgical method. There is no preoperative modality that can differentiate PTA from parathyroid carcinoma. Therefore, the diagnosis of malignancy before surgery is difficult, and the intraoperative findings including histology are important for all cases of parathyroid tumors. Preoperative fine-needle biopsy is also not recommended due to the increased risk of dissemination and inferiority in differentiation between adenoma and carcinoma [3, 5]. (^99m^Tc)-sestamibi scintigraphy is recommended as the first-line imaging method, and ultrasound is recommended as a complementary and hopefully confirmatory examination for preoperative localization. With the conformity of both modalities, the positive predictive value for correct localization of the parathyroid tumor is 97% [18]. In addition, ultrasound findings in the form of calcification and high vascularization may indicate parathyroid carcinoma. However, the presence of a thick capsule is most likely suggestive of parathyroid adenoma [12]. In this case, the presence of a thick capsule with minimal internal flow in the Doppler images reduced the likelihood of malignancy. (^99m^Tc])-sestamibi scintigraphy is based on the accumulation in cells with abundant mitochondria, which display slower wash-out compared to cells with fewer mitochondria, in this way differentiating such cells. Both sporadic parathyroid adenoma and parathyroid carcinoma have such characteristics [12]; therefore, (^99m^Tc)-sestamibi cannot differentiate between benign and malignant tumors. However, in cases of metastatic carcinoma, which accounts for 10–30% of parathyroid carcinoma [14], multiple foci can be detected using scintigraphy [12]. In this case, no other foci on the neck or in the mediastinum were detected.

In the current case, the course of treatment included surgery and follow-up, which were without complications. After approximately 18 months of follow-up, the patient was seen at the endocrinology outpatient clinic with normal calcium and PTH levels, in line with another reported giant parathyroid adenoma [3].

In addition to reporting an extremely rare case, we demonstrated that a very high PTH level might be present in a hyperfunctional giant parathyroid adenoma in a young patient, which can be challenging to differentiate from parathyroid carcinoma. However, in the case of negative familial disposition to MEN1, only mildly increased albumin-corrected calcium levels, lack of nephrolithiasis or osteoporosis, and the presence of a thick capsule on UL, benign adenoma is more likely than carcinoma in the presence of severely elevated PTH.

## Data Availability

No underlying data was collected or produced in this study.
